# Temporal histological changes in lacrimal and major salivary glands in mouse models of Sjogren’s syndrome

**DOI:** 10.1186/1472-6831-13-51

**Published:** 2013-10-05

**Authors:** Jingxiu Xuan, Long Shen, Kishore Malyavantham, Oleh Pankewycz, Julian L Ambrus, Lakshmanan Suresh

**Affiliations:** 1 Department of Medical Immunology, Sichuan University, 610041 Chengdu, China; 2 Division of Allergy, Immunology and Rheumatology, Department of Medicine, School of Medicine and Biomedical Sciences, State University of New York, 14203 Buffalo, NY, USA; 3 Department of Surgery, Medical Director Kidney and Pancreas Transplantation Erie County Medical Center, 14215 Buffalo, NY, USA; 4 IMMCO Diagnostics Inc., 60 Pineview Drive, 14228 Buffalo, NY, USA

**Keywords:** Sjogren’s syndrome, Major salivary glands, Lacrimal gland, Inflammation, Histology

## Abstract

**Background:**

Evidence in imaging studies suggests that there may be differences in glandular involvement in Sjogren’s syndrome (SS) depending on the stage of the disease. No detailed histological studies are available to show if there are any such difference in glandular involvement at various time periods and stages of SS. This cross sectional study examines the inflammatory changes in mouse models of SS at various ages.

**Methods:**

The histological changes in major salivary and lacrimal glands were studied at ages of 3, 6, 9, 12, 15 and 18 months in both sexes in well characterized mouse models of SS, non-obese diabetes mouse and Interleukin-14 alpha-transgenic mice.

**Results:**

Our results indicate that early inflammation concurrently occur in submandibular and lacrimal glands around the age of 6 weeks. Parotid glands are involved much later in the course of SS with less severe inflammation. Sublingual glands are rarely involved.

**Conclusions:**

Our conclusions are that SS may be an organ specific disease with early inflammation occurring in submandibular and lacrimal glands, followed by the parotid. Non organ specific events occur in later courses of the disease. The understanding of the disease progression is important in tailoring early local therapeutic interventions before complete destruction of salivary and lacrimal glands.

## Background

Sjogren’s syndrome (SS) is an autoimmune mediated disorder characterized by inflammation and dysfunction of salivary and lacrimal glands resulting in dryness of the mouth and eyes [1]. The etiology and pathogenesis of SS is not well understood. The diagnosis of SS is complex and based on multiple criteria encompassing serology, histopathology, and imaging [1]. The histological hall mark of SS is foci of periductal and perivascular inflammation of lymphocytes mainly in the salivary and lacrimal glands. The inflammatory infiltrate leads to complete destruction of acini with loss of secretory function of the glands and eventually development of B-cell lymphomas [2,3]. All of the diagnostic criteria, in addition to the recently revised American College of Rheumatology classification recommends use of labial minor salivary gland (MSG) biopsy exhibiting focal lymphocytic infiltrate for diagnosis of SS [4,5].

The clinical changes of dry mouth and dry eyes are predominantly due to destruction of major salivary glands and lacrimal glands. There is evidence in the imaging studies using scintigraphy, magnetic resonance imaging and ultrasonography showing that salivary and lacrimal glands display changes associated with SS. The imaging studies also show that there may be differences in the progression of disease in the different exocrine salivary and lacrimal glands. The submandibular glands show the most diagnostic alterations regarding salivary flow rates in SS causing the dry mouth, followed by parotid [6-8]. The sublingual glands are less severely involved in SS than the other major salivary glands [9]. Imaging and histological studies of lacrimal gland are minimal and available results show that there may be progressive dysfunction of the gland [10].

Although there have been evidence in imaging studies showing the salivary gland changes in SS depending on the stage of the disease, there have been no cross-sectional and temporal histological studies available. The reasons are that it is difficult to study the longitudinal changes occurring in the major salivary glands and the lacrimal glands in humans due to the inability to obtain serial biopsies. The mouse models of SS provide the ideal opportunity for histological studies because of several advantages. The mouse models replicates the disease course in humans, clinical features and complications of SS and gives researchers insights into the disease pathogenesis. Other salient features of mouse models that are of advantage in the study of the SS are the short lifespan of a mouse allowing to study changes occurring from birth to death, uniform genetic backgrounds, and relative ease in breeding of the models [11,12].

The aim of the study is to describe the histological changes occurring at various ages (temporal) in the major salivary glands (submandibular, parotid and sublingual) and lacrimal glands of the mouse models. We selected non-obese diabetes (NOD) mouse, the most popular and extensively studied mouse model of SS [13,14] and interleukin-14 alpha-transgenic (IL-14α-TG) mice, the mouse model for primary SS [15,16]. A control group was also included in the study.

## Methods

NOD mice and C57/BL6 (control) mice were obtained from the Jackson Laboratory (Bar Harbor, ME) and housed in the Laboratory Animal Facility at the State University of New York at Buffalo. IL14-α-TG mice were made by Dr. Ambrus’ laboratory and maintained in the Laboratory Animal Facility at SUNY Buffalo [17]. Animal studies were approved by institutional animal care and use committee at the State University of New York at Buffalo. All procedures were conducted in compliance with the institutional guidelines on use of animals in research.

A total of 108 mice were included in the study, 2 male and 4 female mice for each group (C57/BL6 controls, IL-14α TG and NOD) were sacrificed at time points ranging from 3 to 18 months (6 time points of 3, 6, 9, 12, 15 & 18 months). The major salivary (parotid, submandibular, & sublingual) and lacrimal gland were removed, bilaterally, and placed in 10% formalin and tissue processed for hematoxylin and eosin staining (H & E) for evaluation using standard processing techniques. Three 5 μm thick embedded paraffin sections separated by 50 μm were used for the analysis. The sections were reviewed and scored blindly by a pathologist using the classification criteria described below.

**Figure 1:**
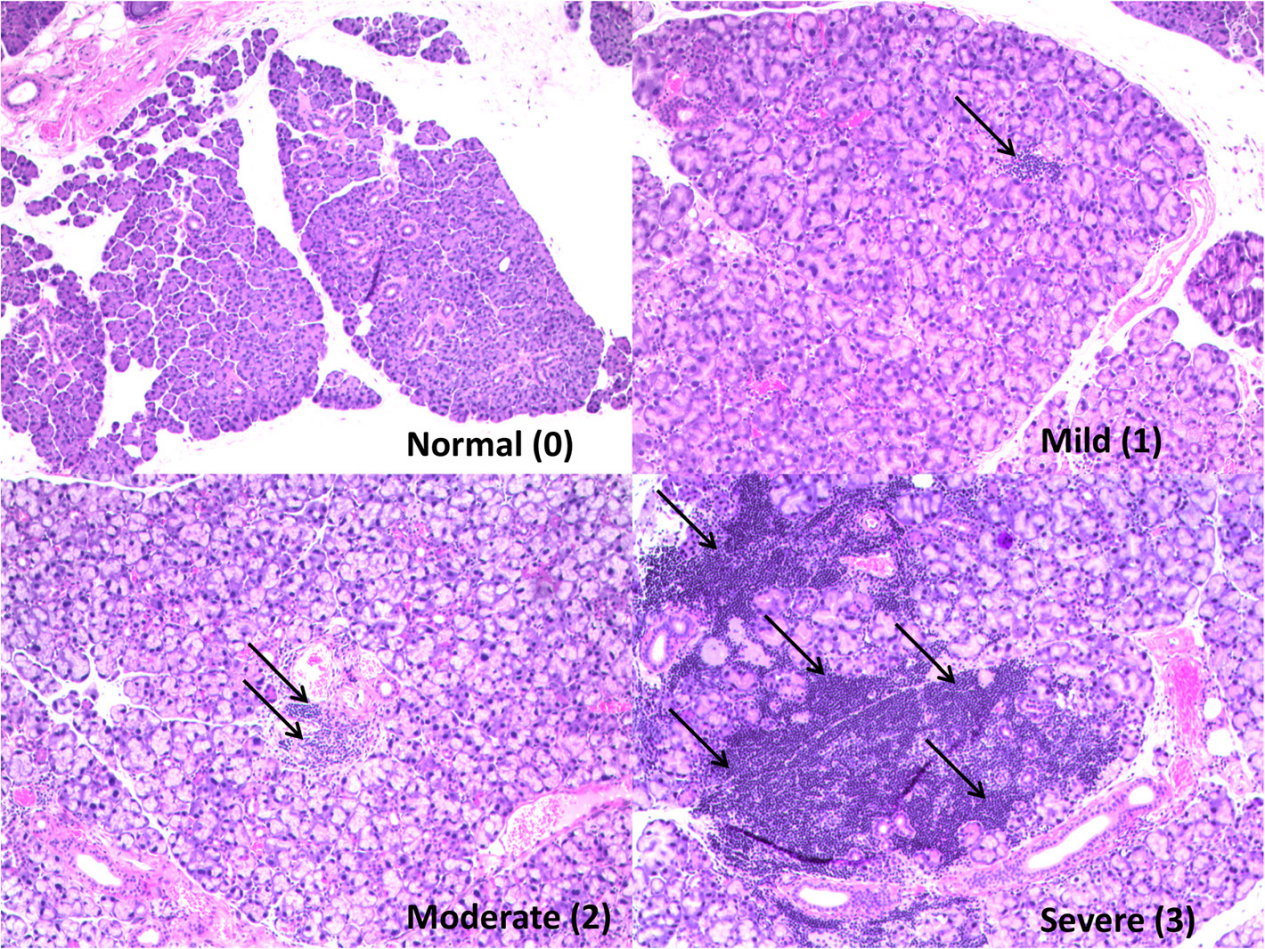
**Representative sections from submandibular glands of IL-14α-TG mouse depicting normal, mild, moderate and severe inflammation (H & E, Original Magnification × 40).**

The extent of observed inflammation was categorized into 4 different levels and assigned an inflammation score of 0 to 3 (Figure 1). A score of 0 indicates no detectable levels of inflammation by histological analysis; a score of 1 indicates mild inflammation but no foci; a score of 2 indicates moderate levels of inflammation and one foci (A foci was defined as consisting of ≥50 mononuclear cell/mm^2^); Score of 3 indicates severe inflammation and more than one foci [18].

Statistical method: the data were analyzed by Chi-Square test, χ^2^ > χ^2^_(1, 0.05)_. The P value <0.05 was considered statistically significant. When the sample size was less than 40, Fisher exact probabilities were utilized.

## Results

**Figure 2:**
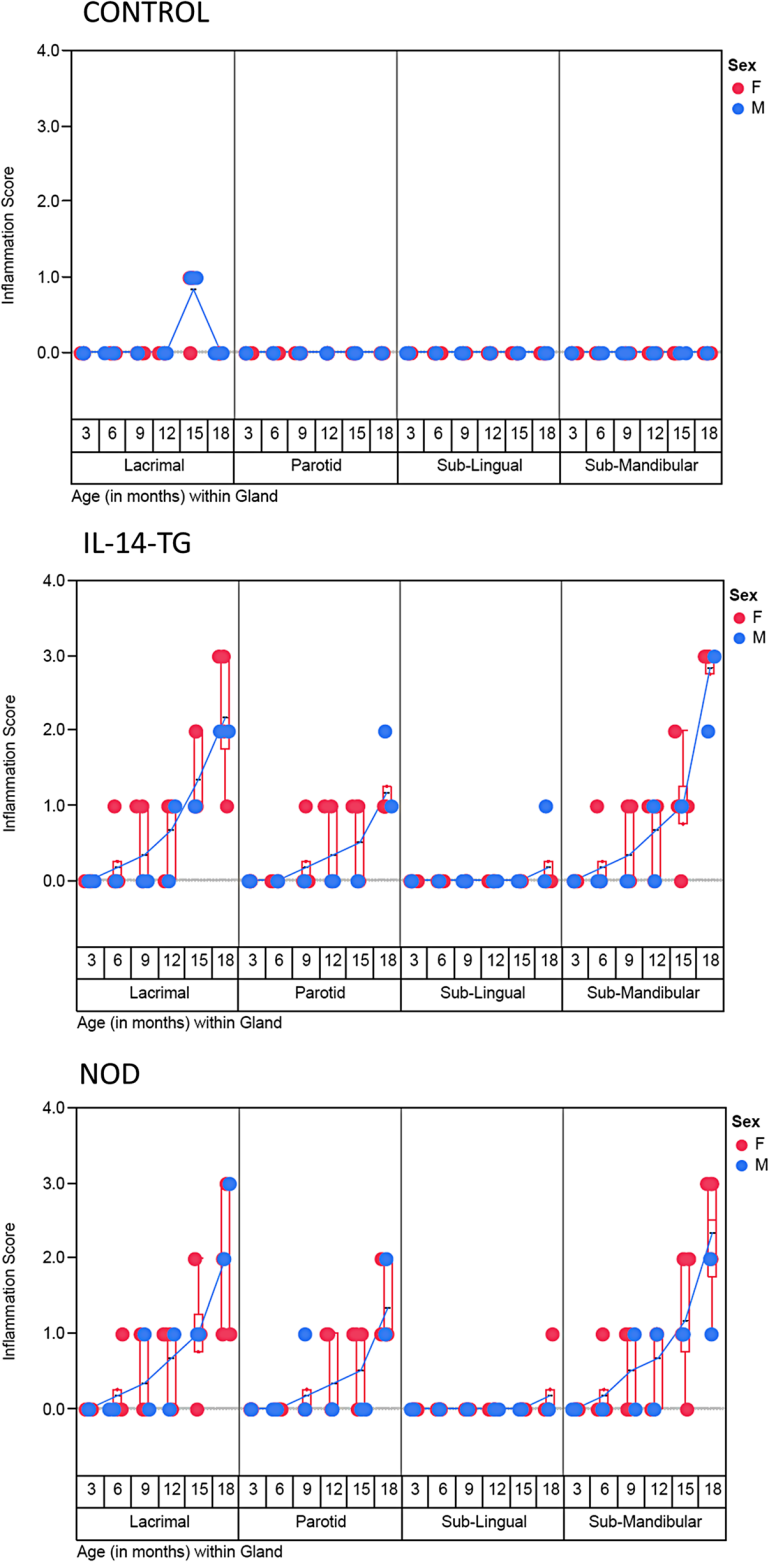
**A scatter plot of inflammation score on Y axis was plotted against the type of gland grouped with age of the mice (in months) on X-axis.** The progression of inflammation from 3 to 18 month time point can be observed for each gland and category of mice. Red and Blue circles indicate Female and Male Mice respectively. A blue line connects the mean value of inflammation score for each time point and gland. The data points (n = 6 for each time point/gland/type of mice) are jittered but a box plots (red) have been provided to depict the interquartile range and distribution for the observed levels of inflammation.

A total of 108 mice were studied and Figure 2 summarizes the 864 observations. All mouse glands exhibited similar level of inflammation in the glands on the right and the left side. Therefore the delineation of the sides has not been done for this study. The control mice did not show any inflammation in the major salivary glands of any time points. Interestingly mild inflammation was observed in the lacrimal glands of 5 out of 6, 15 month old control mice. However, no inflammation was observed in the lacrimal gland of the 18 month old control mice.

IL-14α- TG and the NOD mouse showed similar inflammation patterns, in the type of glands affected, the severity and the time point at which it occurred (Figure 2). The mild inflammation of the submandibular and lacrimal glands of the IL-14α-TG and the NOD mouse begins at 6 months of the age. The inflammation becomes moderate at 15 months of age and severe at 18 months of age, when 6 out of 6 mice demonstrated some level of inflammation.

The parotid glands involvement is widespread by 15 and 18 months and relatively less severe compared to the lacrimal or submandibular glands. The sublingual gland involvement is minimal, appears later at 18 months, and the inflammation is less severe.

**Figure 3:**
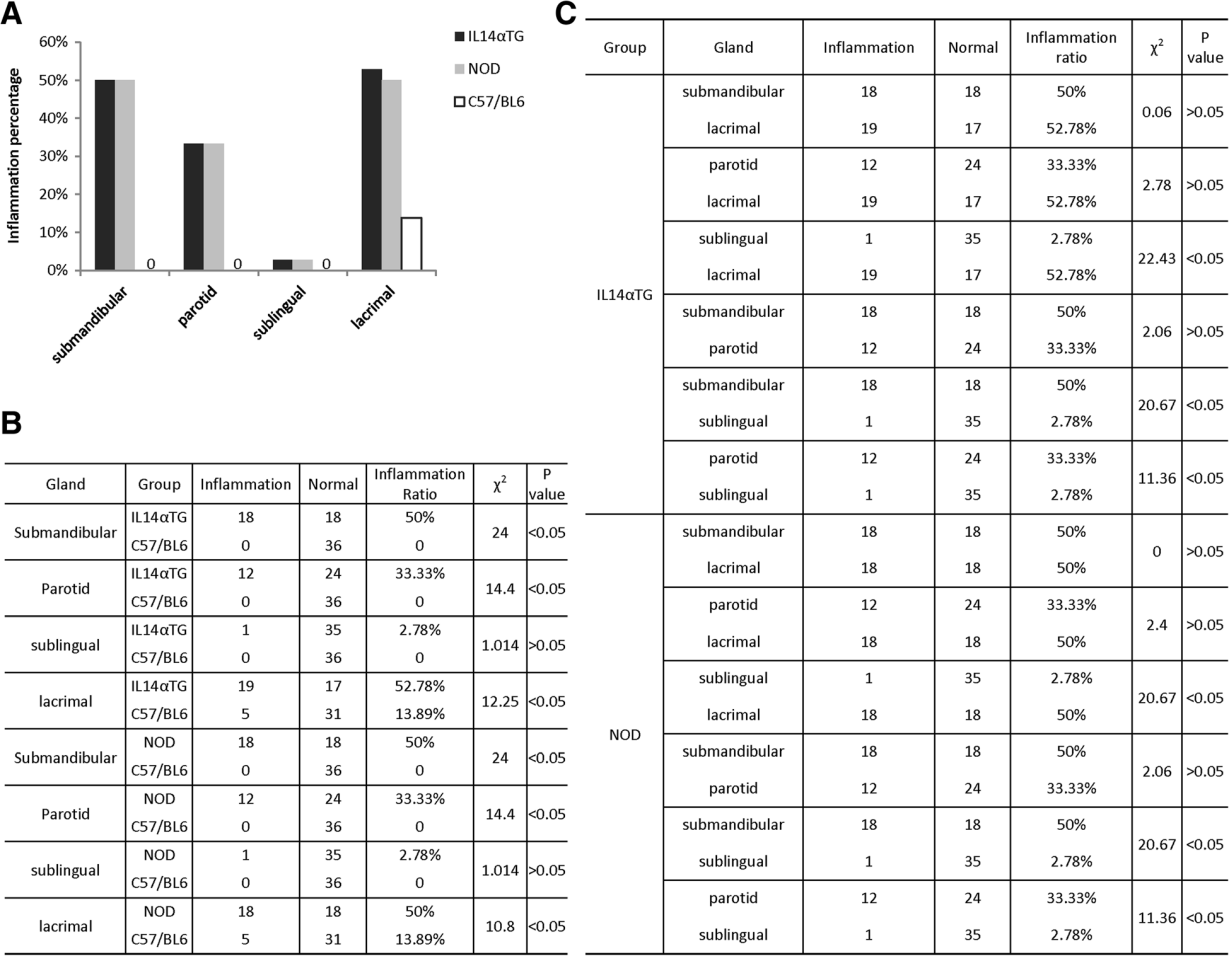
**In A, the inflammation percentage as Y axis, and gland type as X axis, the black column, grey column and black vertical rectangles without filling represent inflammation ratio in the different glands of IL14-α-TG, NOD and C57/BL6, respectively.** For no inflammation in major salivary glands of C57/BL6, they were labeled 0 in **(A)**. According to the amount of inflammation of the different glands in three groups of animal models (n = 36/group, age 0-18 months), the statistical significances of every two group are showed by χ^2^ and P values **(B)**. The differences of inflammation incidences in type of glands are also analyzed using χ^2^ and P value **(C)**.

**Figure 4:**
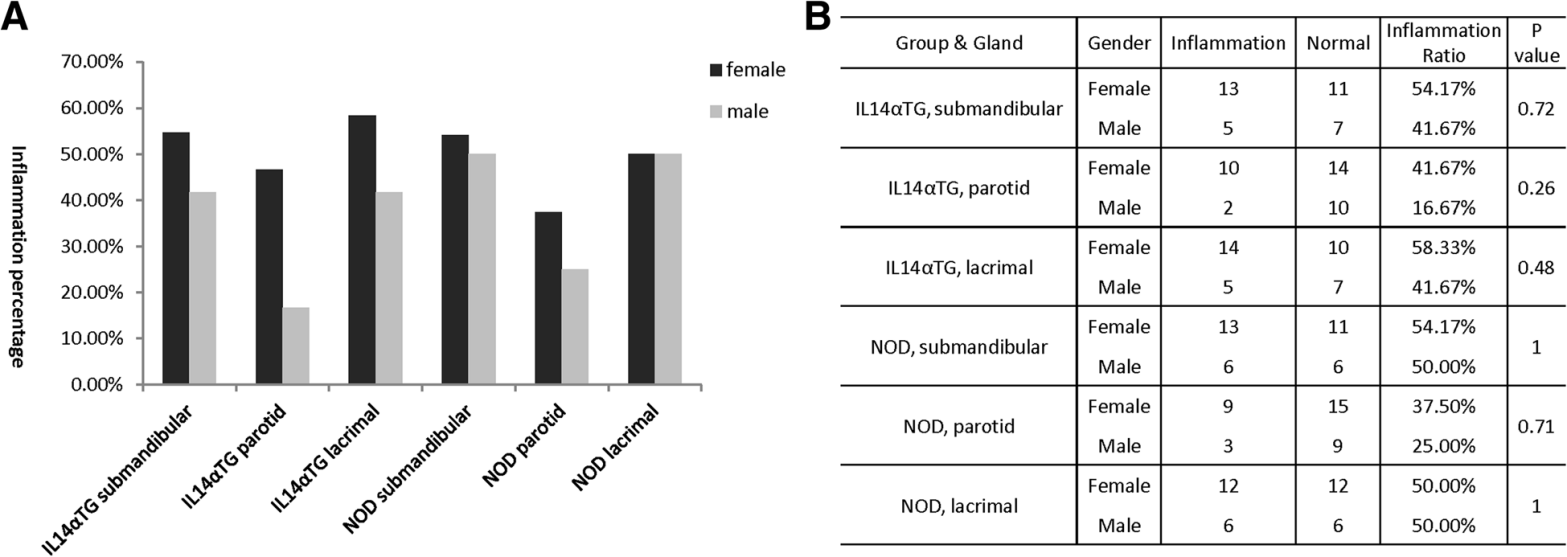
**In this study, we observed the inflammation incidences in gender including the two groups of IL14-α-TG and NOD.** The inflammation ratio of female mice was depicted by black vertical rectangles, and male mice were described by grey ones in inflammation percentage on Y axis against grouped type of glands on X axis (**A**, 24 of Female/group, 12 of Male/group, age 0–18 months). P values of inflammatory mice between female with male were analyzed by Fisher exact probabilities **(B)**, all P values > 0.05 as shown, no significant difference based on gender.

In Figure 3A, the data showed that out of 36 IL-14α-TG, inflammation was noted in 18 submandibular glands, 12 parotid glands, 1 sublingual gland, and 19 lacrimal glands between 0 to 18 months of age. The incidences of inflammation were 50%, 33.33%, 2.78%, and 52.78% respectively. NOD mice demonstrated similar inflammation as the IL-14-αTG mice. While the control group of 36 C57/BL6 mice had no inflammation in major salivary glands, and only had 5 in lachrymal glands, the overall incidence of inflammation was 13.89%. The difference in inflammation between IL-14α-TG mice or NOD mice and the control C57/BL6 mice was statistically significant. (P < 0.05, Figure 3B). When comparing IL-14α-TG and NOD mice, there was no statistically significant difference when evaluating either the submandibular, parotid or lachrymal glands (P > 0.05). There was statistically significant difference between the sublingual gland and either the submandibular, parotid or lachrymal glands (P < 0.05). Inflammation occurred in the submandibular, parotid and lachrymal glands (Figure 3C). There was no statistically significant difference in inflammation in any of the glands when comparing male and female mice in both the NOD and IL-14α-TG strains (P > 0.05, Figure 4A, 4B).

## Discussion

The NOD mouse is one of the popular mouse models for the study of SS. In addition to having diabetes shows features of SS similar to human such as dry mouth, dry eyes, presence of autoantibodies (Ro, La), lymphocytic infiltration of salivary, lacrimal glands and hypergammaglobulinemia [13]. The chronological and histological changes occurring in submandibular gland has been well studied in the NOD mice. The initial changes start appearing at approximately 8 weeks-old [14]. All of the NOD mouse sub mandibular glands are affected by age 18 months [11,14,19]. However, no chronological studies on inflammation of parotid, sublingual or lacrimal glands for the NOD mouse or any other mouse model are available.

The Interleukin-14 alpha-transgenic mouse model could be an ideal mouse model to study primary SS. Interleukin-14 [20] increases germinal B-cell proliferation, including B1 and B2 activated cells [20]. The IL-14α-TG reproduces the features of primary SS as seen in patients in the same relative time frame as humans, including autoantibodies, early destruction and infiltration of the salivary and lacrimal glands with lymphocytes, and eventually lymphoma [15,16,21]. The mouse also develops inflammation of other organs such as kidney and lungs by 15 months of age [15,16].

The major salivary glands, parotid, submandibular and sublingual glands contribute to over 90 percent of the saliva secreted [22,23]. The minor salivary glands scattered throughout oral mucosa contribute to rest of the 7–10 percent of saliva [24,25]. Parotid gland secretions are purely serous in nature and contribute to the majority of stimulated saliva [26]. The submandibular saliva is mixed in nature containing mucus and serous secretions. The unstimulated saliva is mainly produced from submandibular gland [26]. The sublingual saliva is predominantly mucus and its contribution to unstimulated and stimulated whole saliva is limited. The most diagnostic alterations in SS regarding salivary flow rates occur in submandibular gland followed by the parotid [7,27]. The results of our studies show that in addition to salivary flow changes, early inflammatory infiltration is seen the submandibular glands of both NOD and IL-14α-TG mouse. The inflammatory change occurs as early as 6 weeks of age and is most severe during the 16 weeks of age. The parotid glands inflammatory changes occur later at 12 weeks of age. The sublingual glands are rarely involved in the both mouse models (Figure 2).

The lacrimal gland changes of lymphocytic infiltration in our study occurs approximately at 6 weeks of age onwards and occur in tandem with submandibular gland inflammation (Figure 2). The changes appear in both the NOD and IL-14α-TG mouse models. Earlier studies of lacrimal glands of NOD mouse have shown that the inflammation occur between 6–8 weeks of age [28]. There are gender-related differences in the degree of exocrine gland inflammation that have also been reported in NOD mice [29]. This difference in severity of inflammation in the lacrimal glands of the NOD mouse may be attributed to hormonal influences of insulin. The deficiency of insulin in NOD mouse causes decrease in activity and survival of lacrimal gland acini [30].

In one of the few comparative histological studies available comparing between lacrimal and submandibular glands, in the NOD mouse model, the inflammation was found to be higher in the lacrimal, as compared to submandibular glands [31]. Interestingly there was difference in inflammation between the male and female mouse. The inflammation was worse in lacrimal glands of males than females [32]. Studies also show that inflammation began at around 6–8 weeks of age. By the 12th week approximately 25% of the lacrimal gland was infiltrated with lymphocytic inflammation. There is evidence that androgens may play a role in inflammation. The orchiectomy of NOD mice decreased lymphocytic inflammation due to lack of androgens [29]. There was no significant difference between the male and female mouse in our study.

Although major salivary and lacrimal glands show saliva or tear flow and inflammatory changes in early SS, there have been only a few studies showing their utility in diagnosis. The studies using parotid [33,34] or sublingual gland [35,36] biopsies have shown that they may provide better specificity and sensitivity for diagnosis of SS than the commonly used minor salivary gland biopsies [34]. To our knowledge, no studies of submandibular or lacrimal gland biopsies for diagnosis of SS have been reported in the literature. The reasons that there is a lack of major salivary or lacrimal gland biopsies may be due to the invasive nature of the surgical procedure and requirement of general anesthesia in comparison to minor gland biopsies. In addition, complications such as altered sensation in the pre-auricular area, sialocele, facial nerve damage, salivary fistula and swelling in the floor of the mouth may also occur in the major salivary gland biopsies [27].

Recent evidence suggests that the early disease of SS in the mouse models and the humans may be organ specific. In both patients and animal models there the injury to the salivary glands occurs prior to and independent of parenchymal lymphocytic infiltration [37]. The injury is followed by local production of soluble lymphotoxins in the salivary or lacrimal glands of IL-14α-TG mice that is necessary for the development of overt SS [15]. In addition, the antibodies to salivary protein 1 localized to major salivary glands and lacrimal glands, are produced early in the disease, before the production of antibodies to Ro La, and/or lymphocytic infiltration of the minor salivary glands [21].

## Conclusion

In conclusion, our findings indicate that the early inflammation in male or female mice concurrently occurs in the submandibular and lacrimal glands at around the age of 6 weeks. The parotid glands are involved much later in the course of SS with less severe inflammation. The sublingual glands are rarely involved. Based on our mouse model studies, early SS begins as an organ specific disease in the submandibular and lacrimal glands and progresses to the other salivary glands. Later during the course of the disease other organ systems are affected and development of lymphoma. Based on these findings, we hypothesize that human SS may mimic the mouse model and identification of organ specific antibodies, in the major salivary and lacrimal glands may lead to early diagnosis and thereby treatment of SS.

## Competing interests

The authors declare that they have no competing interests.

## Authors’ contributions

LS, JA, LgS: Conceived the study and participated in its design and coordination. JX, LgS, LS and OP were involved in the maintenance of the mouse models, sacrifice of the mouse, and harvest of the tissues. JX, LS and JA performed the histological analysis. JX, LS and KM were involved analysis, graphs, tables, and photomicrographs. JX, OP, KM and LgS performed the statistical analysis. All authors read and approved the final manuscript.

## Pre-publication history

The pre-publication history for this paper can be accessed here:

http://www.biomedcentral.com/1472-6831/13/51/prepub
